# Stepping Stones and Creating Futures: A group‐based approach to addressing violence against women through working with men

**DOI:** 10.1002/jclp.23293

**Published:** 2021-12-16

**Authors:** Andrew Gibbs, Smanga Mkhwanazi, Yandisa Sikweyiya

**Affiliations:** ^1^ Gender and Health Research Unit South African Medical Research Council Pretoria South Africa; ^2^ Centre for Rural Health, School of Nursing and Public Health University of KwaZulu‐Natal Durban South Africa; ^3^ School of Public Health University of Witwatersrand Johannesburg South Africa

**Keywords:** gender transformative, group sessions, poverty, violence

## Abstract

In low‐ and middle‐income countries, group‐based interventions to address intimate partner violence (IPV) working with men, whether or not they are violent themselves, are increasingly common. Stepping Stones and Creating Futures (SSCF) is one intervention demonstrating reductions in men's perpetration of IPV through working with men around gender inequalities and livelihoods. Using a case study of Thembani, a young man living in an urban informal settlement in South Africa who was a participant within a large randomized controlled trial evaluating SSCF, we discuss how his use of violence changed. This reduction occurred through recognition that his situation was not a personal failing, but similar to others, thus reducing the shame he felt, learning to control his anger, and starting to understand how others felt when he used his power over others. This case study provides some initial evidence about how group‐based interventions working with men may start to transform men's practices.

## 1 INTRODUCTION

In South Africa, men's perpetration of intimate partner violence (IPV) is relatively common among the general population. In a population‐based representative household sample from Gauteng Province, 56% of men reported ever perpetrating physical IPV against a partner, while a third (31%) reported lifetime perpetration of sexual IPV (Machisa et al., 2016). This is exceedingly high, and confirmed previous population‐based samples in KwaZulu‐Natal and the Eastern Cape Provinces, which found 29.7% of men reported perpetrating rape in their lifetime (Jewkes et al., 2011). While men's perpetration of IPV may be common, in general, it is particularly acute in urban informal settlements (slums) settings, likely driven by the social context and more youthful population. Among a convenience sample of young (18–30 years) men in Durban, KwaZulu‐Natal Province, 57% reported past‐year physical and/or sexual IPV perpetration (Gibbs et al., 2018) and this was a similar prevalence to a convenience sample of men in an urban informal settlement in Gauteng Province (Christofides et al., 2020).

Underlying these high rates of IPV perpetration are an interlocking set of factors: poverty, gender inequitable masculinities, and inequitable gender norms (McCarthy et al., 2018), the acceptability of violence in everyday social relationships, men's own experiences of violence, and adverse events in childhood and later life, and poor mental health, including substance use (Gibbs et al., 2018; Gibbs, Dunkle, Ramsoomar, et al., 2020; Jewkes, 2002; Spencer et al., 2019). These are all likely exacerbated by the contexts of urban informal settlements, which have heightened poverty, substance use, and community violence. Thus, men's violence is at heart a social and economic problem, reflecting a social determinants approach to health, and mental health (Lund et al., 2014).

Recognizing how IPV perpetration is not simply an individual mental health issue but is located and sustained by wider social and economic systems and contexts, a significant focus of IPV prevention in the global South has been working with men, whether or not they perpetrate violence themselves, rather than focusing on perpetrators only. This approach has moved away from information provision, or role‐model approaches, towards actively supporting men to change the way they see themselves as men, and the practices linked to these ideas (Jewkes et al., 2015). Broadly, these interventions are termed “gender‐transformative” and work with small groups of men (and often women in separate groups) to address the multiple overlapping factors that drive IPV in each specific context (Jewkes et al., 2015).

This article provides a case study of one group‐based gender‐transformative intervention, Stepping Stones and Creating Futures (SSCF), and the experience of one young man going through the process.

## 2 DESCRIPTION OF SSCF

SSCF comprises two separately developed manuals, which provide a series of participatory activities for groups of approximately 10–20 young men (and women) to work through, led by a trained peer facilitator. In total, there are 21 sessions, with each session lasting approximately 3 h. Stepping Stones were originally developed to address HIV‐AIDS, sexual health, gender inequalities, violence, and communication skills, in Uganda in the 1990s (Welbourn, 1995). Stepping Stones have been adapted and implemented widely. In South Africa, stepping Stones were adapted and evaluated and became a 10 session intervention (Jewkes et al., 2010). Creating Futures is 11 sessions and was developed in South Africa to address issues related to the high levels of poverty in urban informal settlements and to build onto the Stepping Stones manual. It draws on a sustainable livelihoods framework (Misselhorn et al., 2014), and includes sessions on livelihoods, and making and keeping work—see Table 1 for a full outline of the combined interventions. The intervention includes women and men in separate groups and assumes heterosexual participants.

**Table 1:** Manual outline for Stepping Stones and Creating Futures

Stepping Stones	Aim of session
Session A: Let's communicate	To help a peer group form itself. To help participants develop skills of listening and analysis of communication and cooperation
Session B: How we act	To help participants explore images and realities of the ideal man and woman—how these are shaped by the actions of all of us and the implications this can have for the individuals concerned
Session C: Sex and love	A first look at images of sex and sexual health problems and an exploration of what we look for and give in love
Session D: Conception and contraception	To explore problems and concerns about conception and contraception
Session E: HIV	To explore our knowledge about HIV and “safer sex”
Session F: Safer sex and caring in a time of AIDS	To continue our discussions about safer sex and to familiarize participants with the use of the condom and other forms of contraception
Session G: Gender violence	To explore violence in relationships and sources of support and help
Session H: Let's support ourselves	To find new skills to change the ways in which we behave
Session I: Let's assert ourselves	To develop more assertive skills
Session J: Let's look deeper	To study why we behave in the ways we do
Creating Futures	
Session 1: Introduction and storytelling	To explore participants' course expectations To support participants to reflect on their life stories
Session 2: Situating the self	To support participants to reflect further on their life stories to reflect on the resources they draw on in building their lives and livelihoods To facilitate participants' identification of some medium‐term goals for their livelihoods
Session 3: Resources needed to sustain livelihoods and reach goals	To create awareness with participants about the resources that people need to: produce livelihoods; cope with crises in their lives; and work towards their identified goals
Session 4: Social resources	To increase participants' awareness of how to build and maintain reciprocal relationships of trust (inside and outside the community) that assist in improving their lives To enhance participants' understanding of the advantages and disadvantages of community participation and how to draw benefits from community participation To support participants to identify the role of social resources in reaching the livelihood goal selected in Session 1
Session 5: Peer group meeting	To enable the male and female groups to share how their livelihood goals and aspirations, and their views and experiences of gender norms and pressures, influence their sexual experiences
Session 6: Education and learning	To enhance participants' ability to recognize that there are multiple ways of learning, including experiential and educational, formal and informal To encourage participants to identify strategies to identify, utilize and build on learning opportunities To enhance participant's ability to critically assess how they decide what determines their own success
Session 7: Getting and keeping jobs	To enhance participant ability to reflect critically on work expectations and on own behaviors that impede or increase the ability to get a job and to keep appropriate work opportunities and increase their own ability to market their own skills and apply for work To support participants to come up with practical strategies in overcoming challenges in job seeking and maintaining a job
Session 8: Income generating activities	To enhance the ability of participants to identify viable, accessible business opportunities, and the resources necessary to respond to such opportunities To enhance the ability of participants to identify basic business principles, including business risks
Session 9: Saving, and coping with shocks (Part A)	To motivate critical thinking among participants around spending patterns and strategies for saving To support participants to explore causes and consequences of getting into debt and ways of overcoming debt
Session 10: Saving, and coping with shocks (Part B)	To enhance participants' ability to identify different types of shocks and crises and the different ways of responding and the impact of responses To create awareness among participants of the role of saving and different ways of accomplishing saving
Session 11: Reflecting on learning and looking ahead	To allow time for participants to reflect on what they have learned through the intervention, and think further about their goals looking ahead

The combination of the interventions, one addressing gender inequalities, the other weak livelihoods, was done to address two concerns. First, a strong argument has been made that men's perpetration of IPV is partially driven by poverty (Hatcher et al., 2020), and addressing poverty, as well as gender inequalities may enhance intervention outcomes. Second, working on men's livelihoods may be an important “draw‐card” for men's involvement in violence prevention programs, especially where they struggle to survive. At the time of the intervention, there had been very few public health interventions addressing poverty as a way to reduce men's perpetration of violence (Gibbs et al., 2012), despite these compelling reasons.

When the combined SSCF intervention is delivered by new facilitators, they receive extensive training on the approach. In general, facilitators have been selected from communities similar to those being “targeted” by the intervention. Facilitators tend not to have much prior experience in delivering interventions, and, thus, receive a 5‐week training program. In the first 2 weeks, they experience SSCF as participants, and for the following 3 weeks learn specific information (e.g., about HIV), training on facilitation skills, and practice delivering the intervention. Facilitators are normally supervised by a senior facilitator who observes sessions and provides additional training on a weekly basis.

### 2.1 Rationale/theory

SSCF, like many participatory, gender‐transformative interventions (Gibbs, Vaughan, et al., 2015; Jewkes et al., 2015) draws on Paulo Freire's approach to adult education and learning (Freire, 1973). Thus, it comprises a mixture of skill‐building activities on specific topics (e.g., communication skills), alongside a broader focus on creating “safe social spaces” (Campbell & Jovchelovitch, 2000; Campbell & MacPhail, 2002). Safe social spaces emerge through a process of trust‐building in the group, and open discussion and reflection on important issues in people's lives. Freire (1973) suggests that through this process of discussion, people will start to come to recognize that their experiences are part of a broader pattern of social relationships, rather than individual experiences (and failings), which he referred to as “critical consciousness.”

This process of creating safe social spaces for skills building and discussion, aiming to foster critical consciousness, is the underlying model through which men start to reflect on their situation and leads to behavior change. SSCF works to reduce men's violence in several ways. First, it provides important emotional relief for men, who often have limited spaces for sharing emotions and struggles in life. Second, recognizing their situation is not a personal failing, but connected to a wider process, it supports them to reduce their shame and anger, and provides them with new techniques to deal with anger. Third, it enables men to start to discuss and reflect on issues related to gender dynamics, violence, and relationships, in ways that enable them to experiment with alternative ways of communicating with each other, partners, and other family members. These steps are underpinned by working to address young men's economic position, which supports them to think about how to make a basic living given the current economic situation, as well as providing them a foundation for change.

### 2.2 Evidence base

Stepping Stones and Creating Futures has been subjected to one large randomized controlled trial (RCT) in urban informal settlements in eThekwini Municipality, KwaZulu‐Natal Province, South Africa with men (and women) aged 18–30 years. In this trial, we recruited 338 men into the intervention arm, and 336 men into the control arm and followed them up over approximately 24 months. Participants were recruited through community meetings, and spending time in the communities to identify potential participants. Participants were not in formal work or education, aged 18–30, and normally resident in the community. Baseline data were collected before the intervention. The intervention was delivered to men over about 3 months. We collected data from all men we could contact after 24 months after the baseline was completed, finding three‐quarters (75%) of original participants. Analysis by intention to treat showed the outcomes of the trial for men were positive: IPV perpetration by men was significantly reduced 24 months after baseline (approximately 18–21 months after the final session); there was an indication of a reduction in the perpetration of non‐partner rape, and alcohol use was also significantly reduced; finally, men's livelihoods were also significantly improved (Gibbs, Washington, et al., 2020). For women in parallel groups, there was no impact on their experience of IPV (they were not in relationships, in general, with the men who participated in the trial), but they did report improved livelihoods. The control group received nothing during the study period but was provided with the intervention once the final data had been collected. Full details of the trial are available elsewhere (Gibbs, Washington, et al., 2020).

While overall positive results were seen there were challenges for the intervention. A key one was men's attendance, with just under half (42.9%) of men recruited into the trial attending only 0, 1, or 2 sessions and a further third (38.3%) attending between 3 and 15 sessions. Men who attended few sessions had lived for a shorter time in the community, and were more likely to have worked in the past three months at baseline (Gibbs, Dunkle, Washington, et al., 2020). Those who attended more sessions were also more likely to reduce their violence perpetration.

The SSCF trial complemented a previous evaluation RCT of Stepping Stones alone (i.e., without the inclusion of Creating Futures) by a team from the same institution. This prior evaluation was implemented among school‐going women and men (aged 15–26 years) in rural communities in the Eastern Cape Province (Jewkes et al., 2008), and found similar results, with reductions in men's perpetration of IPV, and alcohol use. More widely, there is an emerging evidence base globally, primarily in uncontrolled pilots, where variations of Stepping Stones have also demonstrated reductions in alcohol use and IPV perpetration (Skevington et al., 2013) and recent pilots of an adapted SSCF approach—again by a team from the same institution—have been implemented with households in Tajikistan and Nepal and showed positive trends in violence reduction and alcohol use (Mastonshoeva et al., 2019; Shai et al., 2020).

## 3 CASE ILLUSTRATION

This case illustration draws from the SSCF trial which ran from 2015 to 2018 in eThekwini Municipality. The study received ethical approval from the South African Medical Research Council's Ethics Committee (EC006‐2/2015), and the Biomedical Research Ethics Committee, at the University of KwaZulu‐Natal (BFC043/15) before the study commenced. All participants provided written informed consent for the main trial, and separately for the qualitative research which was conducted with a subgroup of participants. The qualitative data were collected by a trained researcher, who conducted a series of in‐depth interviews with men during and after the intervention. The final interview was conducted about 21 months after the intervention was completed. All identifying information, including the participant's name, has been changed.

### 3.1 Presenting problem and client description

Thembani was 24 years old at enrollment into the RCT of Stepping Stones and Creating Futures (June 2016), living in an informal settlement in eThekwini Municipality. In contrast to many others in the study who had migrated recently from the countryside, he was born in the community he now lived in. He lived with his father and his brother and he also had a sister who did not live with him. He described his childhood positively, apart from the death of his mother, and his father played an important role in his life growing up and currently: “because we have been through a lot, he [his father] helped me and he showed me love, he raised me until I became a man, he also advises me on things.” At school he had done well, completing his final high school exams (matric), which only an estimated third of young men in the trial had, and did well enough to seek entrance into higher education.

Despite Thembani's positive start in life, after completing high school, his forward progression in life stopped. He could not afford fees for higher education and fell ill. He also lost a temporary job. He felt acutely aware of how things had not gone the way he wanted: “it saddens me because it has been 5 years since I completed matric [high school], but there is no success in my life.” He was also ashamed as he was not working and had to rely on his sister to survive and recognized how he and other young men like him were looked down upon: “many [men] are respected. I can say that 40% of them are respected because they are working, then the other 30% are focused on crime. In this place there is a lot of crime. Then the other 30% is based on the fact that maybe they are unemployed then they end up being disrespected in the community if you are unemployed.” Many non‐working men referred to themselves as being positioned as children by others in the communities where they lived.

Thembani had a girlfriend for 4 years, but described this relationship also in terms of ‘stuckness’ and failure to progress forwards because he could not establish a home with her, nor get married because he lacked money: “it saddens her because she hears that I should have been somewhere in life because we have been dating for 4 years, our love is going somewhere but life is at a standstill.” He was also violent at times towards her, describing how he hit her when she refused an instruction from him:

Thembani: This current girlfriend, I said that she must come over, but she did not and so we had an argument. I took that anger and I hit her. In the end I discovered that I was wrong to beat her up because she was really at home but had no way of coming over…

Interviewer: How did you feel after you had beaten her up?

Thembani: I felt bossy and that I had fulfilled my wish for that moment, because I thought that I was right.

### 3.2 Case formulation

Thembani was recruited into SSCF when the team of researchers and facilitators spent two days in his community, introducing the project to young people and talking to them. Beyond broad inclusion/exclusion criteria (ability to provide informed consent, not in formal work or education, aged 18–30 years, and living in the community) it relied on people self‐selecting into the project. SSCF was designed specifically to address the interlinked factors of poverty, violence, and hardship Thembani described.

### 3.3 Course of treatment

Thembani attended an SSCF group with about 12 other men, with two sessions a week delivered from June to September 2016. These were delivered in a small community hall, with a women's group also running at the same time. Reflecting on the group process Thembani emphasized how the group was nonhierarchical, and he felt connected to the others who were involved, describing them as “brothers.” In the space of informal settlements with little in the way of trusting peer relationships, a “brother” denoted someone who one could rely on. This did not happen immediately but was through how the sessions built up trust in the group and started to allow them to share issues of importance.

Interviewer: Okay did you trust the people in your group?

Thembani: At first we were skeptical about trusting each other, but as time went by seeing that even the things I said have not been talked about outside the group, and that trust was built in that way where people were able to talk about the burning issues inside themselves.

Importantly he described how the facilitator ensured that everyone got a chance to speak: “Sizwe [the facilitator] he also made sure that when some people were talking a lot he would suggest others should be given a chance to also share and talk, so that they don't keep quiet because they might also have same things to say.” The content of these discussions was around issues they had not previously had a chance to discuss with others: “There were confidential things shared that could not be discussed outside of the group, but we were able to share.”

Through sharing with each other men started to recognize that they often had very similar trajectories in life. This occurred quite clearly during the “River of Life” activity where they mapped out their personal biographies and challenges:

“…I am not sure who it was who stood up, I think it was Zakele that stood up and talked and then people began to understand this thing [River of Life], and then by the time I stood up and shared my story, where I began and got to grade eleven and failed. And then this and that happened. So others asked to start over again because there were things that they forgot to mention.” This was one topic that was sensitive and men were not able to open up in the beginning, yet when they saw someone else opening up they also began to open up too.

As men, including Thembani, came to recognize that their experience of “stuckness”, feeling ill‐at‐ease in the world and failure, was similar to other young men's experiences, and, therefore, not their fault, this had two impacts. First, it helped reduce his shame about his situation and made him less angry. Second, it supported Thembani to start to deconstruct the idealized world and aspirations that he had for his life, and situate his options within realistic opportunities:

…it's the River of [Life]…it was the one about the obstacles that you have faced in your life since childhood up to a point you can see what you have achieved or not, which people have you risked in your life. … it's like okay fine not everything in your life will be smooth and all that there will be times where you will face difficulties.

SSCF also has substantive role‐playing in groups, which supported reflection on challenging issues. Thembani highlighted the impact of how one role play had impacted his understanding of power in relationships:

What helped me change my life in Stepping Stones? Let me see…it was learning about the Statues of Power. That when people have advantages of power, they don't realise how the other person is who is disadvantaged feels like. I had to think that if I have power and do something, I must also think about how the other person feels about it. That is what I learnt, when I have power what the other person's feeling about my power.

He brought this up a second time as well, specifically in relation to women:

I knew that, okay, there are things that we learnt at Stepping Stones you see, that you find that you are not aware of. You see yourself having power over a woman and then you use that power that you have to oppress her, while you wouldn't enjoy it if that thing is done to you and those people…

SSCF sessions also provided Thembani with concrete skills that supported change in his life. Thembani, like many others, mentioned the skills he had learned around communication, and developing and practicing these skills in the group. These skills were transferred into his everyday life and relationships. The other concrete skills he described were in relation to job seeking, and doing simple things such as developing a clear CV, which got modified for each application, and following up on jobs when no response was received. This was followed by their newly developed skill of being able to identify local resources and people to help them develop other income‐generating opportunities existing in their community.

Thembani did not reflect too much on the role of the facilitator in delivering the sessions, beyond mentioning the facilitator made sure everyone had a chance to speak. Yet, the facilitators were crucial to intervention success and had two complex tasks. The first was overt and focused on challenging men's behaviors that were harmful. The second was covert and involved the facilitators establishing themselves in the eyes of the participants as credible “men”, thus enabling the facilitators to push the participants on challenging topics (Gibbs, Myrttinen, et al., 2020). The centrality of the dynamic between facilitator and participants to intervention success was crucial requiring facilitators to recognize the dynamic, and address and repair this dynamic when it broke down in group sessions (Muran et al., 2021).

### 3.4 Outcome and prognosis

For Thembani, the combination of reflection, discussion on gender and power, reduction in shame and sense of “stuckness” in his life, when combined with concrete skills around communication, and starting to map out steps to change his life and act on them to change in small ways the contexts of his situation, resulted in an improved relationship with his girlfriend, and less violence. As he commented a couple of times throughout follow‐up interviews:

I used to do things out of rage but then the project came and taught me how to control my anger and not have power over any men or women. It is better to discuss it and if you fail, it is better to leave than to beat a woman.

Skills around communication, in particular, were central to how Thembani sought to position himself in his relationship with his girlfriend, as well as with other people after the intervention was completed:

Like when you are dating and you have made the mistakes that you do, you must also realise how much your mistakes hurt her, and how things go. So now I communicate well with her…

Like before, I knew that when a person does wrong they have to be beaten. And so now if a person does a mistake, we can sit down and talk about it.

The changes towards a less aggressive and confrontational approach to relationships Thembani described were noticed by his girlfriend, his family, and others in the community suggesting a wider shift in his way of being, and recognizing the connection between IPV and other forms of violence. For instance, Thembani described a situation where he avoided getting into fights with other men, and others in the community commented on his changes:

Yes there were times where you find that people make remarks that you have grown soft, [that] I am not the same [aggressive man] as before, you know what I mean?

Interviewer: What do they mean when they say you are soft?

Like, let's say we are in a shop buying something and then someone jumps the line and goes to the front, you know? And then you just keep quiet. Or else someone steps on your toes, trying to pick a fight with you and you act differently and then they say: “you are soft, you are not the same as the person you were before.”

Thembani also discussed how his approach to his economic situation changed and he started to value “small steps” to change his situation. For instance, he described receiving money and instead of spending it he tried to make additional money:

So I made this money it was about ZAR500 [approx. GBP25], but since I didn't want to spend it on anything I tried saving it and being a loan shark for a short while. So for those people who over spend money after getting paid, they ask to be lent some of the money and then I add interest on it. I have been trying to make money, even though it has been very small but it was getting me somewhere.

Overall Thembani seemed to describe a shift in his approach towards life, with a sense of forward progression once more. In his last interview, he described his relationship with his girlfriend, which contrasted sharply with how he had described it before the intervention: “now between my girlfriend and I, I see things are looking very bright, like okay fine…we are both committed and we have future plans together.”

Yet things remained challenging for Thembani. While his economic situation improved, he was still not in a formal waged job and felt that this was the only way he could truly “succeed” in life. He also described wanting to leave his community and live somewhere “more peaceful.” Additionally, his relationship with his girlfriend remained conflictual, often because he continued to have multiple sexual partners, which he tried to keep hidden. These limitations of SSCF and the context in which it operated limit individuals' ability to significantly change their lives.

## 4 CLINICAL PRACTICE AND SUMMARY

In settings where IPV is common and is partially driven by high levels of poverty, the acceptability of violence in everyday relationships, gender inequitable masculinities, and poor mental health (Gibbs, Dunkle, Ramsoomar, et al., 2020; Gibbs et al., 2018) working to prevent IPV is best done through working collectively with groups of men (and women). Group‐based approaches to addressing men's perpetration reach more men, and benefit men whether or not they are violent, as they support a wider movement towards gender equity, improved economic wellbeing, and reduced alcohol use. The current case study provides a detailed reading of how Stepping Stones and Creating Futures' works to support young, very marginalized men to reflect on, and change their practices in relation to IPV and also violence in other relationships.

The case study of SSCF has several implications for the practice and delivery of similar models to young, marginalized men. First, the central role and skills of facilitators in delivering these interventions, and enabling groups of men to cohere together needs recognition. Facilitators walk a tight‐rope between building relationships between men, encouraging sharing, and challenging the most egregious behaviors of men without losing “face” (Gibbs, Myrttinen, et al., 2020). Thus, training and support for facilitators, who are often grappling with similar challenges to the men themselves appears important.

Second, in contexts where poverty is a key driver of men's perpetration of IPV, working on addressing men's economic insecurity at the same time as addressing gender transformation is critical, and provides both a “drawcard” to the intervention, as well as providing a foundation for effective IPV prevention (Gibbs, Jewkes, et al., 2015; Gibbs, Washington, et al., 2020). Third, a significant challenge for Thembani, and described in prior research (Gibbs et al., 2019), was that men struggle to change their behavior, or sustain behavior change, in contexts where their friends have not been part of similar interventions. Thinking about how to include men's friends and peers in the intervention may be important. Fourth, the combined SSCF intervention is comparatively long, particularly compared to some psychological approaches to addressing violence, and this is reflected in the low levels of attendance towards the end of the intervention. Developing similar interventions that can be delivered in shorter timeframes remains critical. More widely this approach needs adapting and replicating in other contexts independent of the primary research team to confirm this approach within the field of public health.

Stepping Stones and Creating Futures provides a promising intervention for working with groups of men to address their use of IPV in challenging contexts. Its group‐based approach, rooted in adult education models, as recommended by Freire, enables men to achieve some form of critical consciousness, and in so doing start to envisage alternative ways of being in the world.

### 1 PEER REVIEW

The peer review history for this article is available at https://publons.com/publon/10.1002/jclp.23293

## Data Availability

The qualitative data are not available for sharing with others researchers, due to the inability to ensure the anonymity of participants.
